# Optimal Experiment Design for Monoexponential Model Fitting: Application to Apparent Diffusion Coefficient Imaging

**DOI:** 10.1155/2015/138060

**Published:** 2015-12-28

**Authors:** Mohammad Alipoor, Stephan E. Maier, Irene Yu-Hua Gu, Andrew Mehnert, Fredrik Kahl

**Affiliations:** ^1^Department of Signals and Systems, Chalmers University of Technology, 41296 Gothenburg, Sweden; ^2^Department of Radiology, Sahlgrenska University Hospital, Gothenburg University, 41345 Gothenburg, Sweden; ^3^Centre for Microscopy, Characterisation and Analysis, The University of Western Australia, Perth, WA 6009, Australia

## Abstract

The monoexponential model is widely used in quantitative biomedical imaging. Notable applications include apparent diffusion coefficient (ADC) imaging and pharmacokinetics. The application of ADC imaging to the detection of malignant tissue has in turn prompted several studies concerning optimal experiment design for monoexponential model fitting. In this paper, we propose a new experiment design method that is based on minimizing the determinant of the covariance matrix of the estimated parameters (D-optimal design). In contrast to previous methods, D-optimal design is independent of the imaged quantities. Applying this method to ADC imaging, we demonstrate its steady performance for the whole range of input variables (imaged parameters, number of measurements, and range of *b*-values). Using Monte Carlo simulations we show that the D-optimal design outperforms existing experiment design methods in terms of accuracy and precision of the estimated parameters.

## 1. Introduction

The monoexponential model has been used in many different engineering applications. It is frequently used in modeling biomedical phenomena to estimate biologically meaningful parameters. Its applications in quantitative biomedical imaging include apparent diffusion coefficient (ADC) imaging [1], monitoring metabolic reactions [2], and pharmacokinetics [3]. ADC imaging has a wide range of applications including the classification of brain disorders [4], detection of malignant breast lesions [5], identifying stages of cerebral infarction [6], and diagnostic imaging of the kidney [7, 8], prostate [9, 10], and ovaries [11, 12]. ADC imaging is also used to solve challenging clinical problems such as the differentiation of Parkinson's disease from multiple system atrophy and progressive supranuclear palsy [13].

The usefulness of ADC imaging as a quantitative imaging tool has motivated several studies that have investigated the reliability and reproducibility of ADC estimates [7, 14, 15]. From a mathematical point of view, the variance of the estimated ADC values can be minimized by optimizing experiment design. In the case of ADC imaging, experiment design equates to the choice of the *b*-values applied for measurements and their repetitions. In the case of enzyme kinetics, it equates to the sample collection time (*t*). The range of valid sampling points is determined by the biophysical aspects of the problem at hand. For instance, perfusion contamination at low *b*-values [15, 16] and SNR drop at high *b*-values [1] limit the applicable range of *b*-values. An intuitively appealing experiment design is the equidistant (ED) distribution of sampling points on a valid range of the independent variable (*b* or *t*). The ED experiment design method is widely used in the literature [7, 17–19]. However, many studies use nonsystematic and random experiment designs [9, 20] that can considerably influence the results.

Some studies have tried to find the optimal experiment design by empirically evaluating a variety of experiment designs [17]. In contrast, others have developed a theoretical framework by minimizing the variance of the estimated parameters [21–23]. The former strategy may potentially miss the global optimum because of the discretization of the problem and a nonexhaustive search. On the other hand, studies pursuing the latter strategy are based on the Gaussian noise assumption. The Cramer-Rao lower bound (CRLB) of the ADC value is minimized in [23] assuming a Gaussian noise distribution. Hereinafter, we call this method GCRLB. The optimal experiment design in the GCRLB method (briefly described in Appendix B) depends on the ADC values to be imaged. Thus the optimal design must be revised for different applications and even for imaging different organs. Moreover, in applications where the noise assumption is violated, the GCRLB design becomes suboptimal. In this paper, we develop a theoretical framework for optimal experiment design of monoexponential model fitting problems with less restrictive assumptions on noise distribution. Our Monte Carlo simulations using the proposed design method for ADC imaging show that, in the presence of Rician noise, it outperforms the GCRLB and ED methods. In addition, the proposed design is independent of the imaged parameters and provides more robust results.

The remainder of the paper is organized as follows. The next section elaborates the proposed experiment design method.  Section 3 presents results of extensive evaluations and comparisons. A discussion of different aspects and the potential impact of this work is given in Section 4. Finally the conclusion is presented in Section 5.

## 2. Proposed Experiment Design Method

Without loss of generality, hereinafter we focus on ADC imaging as an example of monoexponential model fitting problems. The model for ADC imaging is given by

(1) $$\begin{array}{c} m=m_{0}\exp\left(\;-bD\;\right), \end{array}$$

where *m* is the measured signal when the diffusion weighting factor *b* is applied, *m*_0_ is the observed signal in the absence of such a weighting factor, and *D* is the apparent diffusion coefficient. The parameters to be estimated are *m*_0_ and *D*. In ADC imaging the parameter of interest is *D*. However, there exist applications in which *m*_0_ is also important such as (6) in [2]. Although mathematically two measurements suffice, in practice *N* > 2 measurements are acquired to maximize precision. Depending on the problem at hand, one is permitted to choose the independent variable (*b* in this case) such that *b*_min_ ≤ *b* ≤ *b*_max_. Log-linear least square fitting is frequently used because of its computational efficiency [18]. It can be formulated as follows:

(2) $$\begin{array}{c} \ln m_{i}=\ln m_{0}-b_{i}D,\;\forall i=1,\ldots ,N. \end{array}$$

For *N* measurements we obtain

(3) $$\begin{array}{c} y=Ax, \end{array}$$

where **y** ∈ *ℝ* ^*N*^ contains measurements (ln⁡*m*_*i*_), **x** ∈ *ℝ* ^2^ contains unknown parameters (**x** = [ln⁡*m*_0_ *D*]^*T*^), and **A** is the design matrix below:

(4) $$\begin{array}{c} A=\left[\begin{array}{cc} 1 & -b_{1}\\ \cdots & \cdots \\ 1 & -b_{N} \end{array}\right]. \end{array}$$

The least squares estimator (LSE) of **x** is given by $\hat{x}={\left(A^{T}A\right)}^{-1}A^{T}y$. The precision of the estimation problem above is dependent on the experiment design **A**. For independent and zero-mean measurement noise (on **y**) with constant variance *σ* ^2^ the LSE is unbiased and has the following covariance matrix [24]:

(5) $$\begin{array}{c} Cov\left(\;\hat{x}\;\right)=\sigma^{2}M^{-1}, \end{array}$$

where **M** = **A** ^*T*^ **A** and is usually called the “*information matrix*.” Optimal experiment design entails making the covariance matrix* small* in some sense. It is usual to minimize a scalar function of the covariance matrix. One design approach is to minimize the determinant of the information matrix (D-optimal design). In this paper, we solve the D-optimal experiment design problem for ADC imaging.

**Remark 1 .**

**Figure 1:**
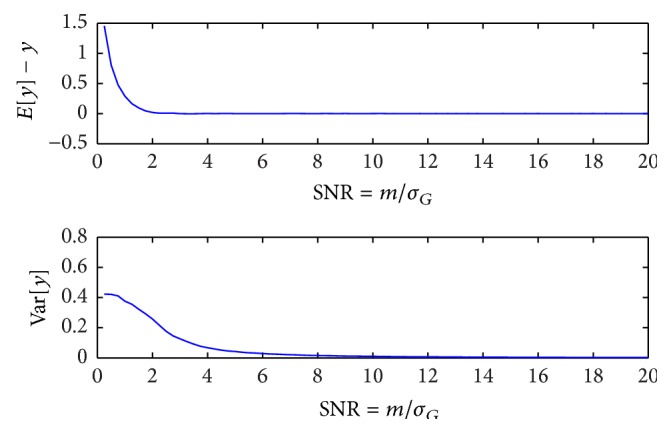
Results of Monte Carlo simulations show that if SNR > 10, then zero-mean and equal variance noise assumptions hold for ADC imaging. Simulation setup: number of Monte Carlo trials *N*_MC_ = 20000, *σ*_*G*_ = 20, and *m* varies from 5 to 20*σ*_*G*_ (equal step size of 5).

The noise distribution on the diffusion attenuated signal (denoted by *m*) is usually assumed to be Rician. To investigate the significance of our noise assumptions in the case of ADC imaging, we use Monte Carlo simulations. Let |*m* + *w*| model the measured diffusion signal, where *m* is the true value of the signal and *w* = *w*_*R*_ + *jw*_*I*_ is the complex-valued measurement noise. The noise components are Gaussian distributed: *w*_*R*_ ~ *𝒩*(0, *σ*_*G*_^2^), *w*_*I*_ ~ *𝒩*(0, *σ*_*G*_^2^). We perform Monte Carlo simulations with Rician noise (on *m*) and the following setup: number of Monte Carlo trials *N*_MC_ = 20000,  *σ*_*G*_ = 20, and *m* varies from 5 to 20*σ*_*G*_ with equal step size of 5. As can be seen in Figure 1, the zero-mean assumption (on *y*_*i*_ = ln⁡*m*_*i*_) holds for SNR > 2 while the equal variance on log-measurements (**y**) holds for SNR > 10. Overall, this shows that both zero-mean and equal variance assumptions hold for SNR > 10. Thus, we expect the proposed method to have diminished performance for high *b*-values and high *D* values.

### 2.1. D-Optimal Experiment Design for Monoexponential Model Estimation

The D-optimal experiment design is based on minimizing the determinant of the covariance matrix of the LSE. The D-optimal experiment design for ADC imaging can be written as follows:

(6) min⁡ det⁡M−1s.t.: M≥0, bmin≤bi≤bmax,i=1,…,N,

where the explicit expression for **M** is

(7) $$\begin{array}{c} M=\left[\begin{array}{cc} N & -\overset{N}{\underset{i=1}{\sum }}b_{i}\\ -\overset{N}{\underset{i=1}{\sum }}b_{i} & \overset{N}{\underset{i=1}{\sum }}b_{i}^{2} \end{array}\right]. \end{array}$$

Noting that minimizing det⁡(**M** ^−1^) is equivalent to maximizing det⁡(**M**) we obtain the following problem formulation:

(8) max⁡ det⁡Ms.t.: M≥0, bmin≤bi≤bmax,i=1,…,N.

### 2.2. Solutions to the D-Optimal Design Problem

The explicit form of the objective function of the optimization problem in (8) is

(9) $$\begin{array}{c} \det\left(\;M\;\right)=N\overset{N}{\underset{i=1}{\sum }}b_{i}^{2}-{\left(\;\sum_{i=1}^{N}b_{i}\;\right)}^{2}. \end{array}$$

It can be seen that, in contrast to previous studies, the D-optimal design is independent of the unknown parameters. Thus, it can be used when imaging different organs as well as in other applications. For *N* = 2 the objective function becomes det⁡(**M**) = (*b*_1_ − *b*_2_)^2^. Therefore the D-optimal design is *b*_1_ = *b*_min_, *b*_2_ = *b*_max_. For *N* = 3 the objective function becomes det⁡(**M**) = (*b*_1_ − *b*_2_)^2^ + (*b*_1_ − *b*_3_)^2^ + (*b*_3_ − *b*_2_)^2^. Consequently, the D-optimal design is *b*_1_ = *b*_2_ = *b*_min_, *b*_3_ = *b*_max_ or equivalently *b*_1_ = *b*_2_ = *b*_max_, *b*_3_ = *b*_min_. Generally, one can see that for arbitrary *N* the D-optimal experiment design is obtained when

(10) bi=bmin∀i=1,…,n,bi=bmax∀i=n+1,…,N,

where *n* = *N*/2 if *N* is even; otherwise *n* = (*N* + 1)/2. In the next section we compare the D-optimal design with the ED and GCRLB designs.

## 3. Evaluation and Simulation Results

In this section we evaluate the proposed D-optimal experiment design method and compare it with existing optimal design methods. We run Monte Carlo simulations using the pseudo-algorithm given in Appendix A. In our simulations we use the Rician noise distribution. While this does not match the noise assumptions of our theoretical framework, it permits a more realistic evaluation of the results for ADC imaging. We use the range [0.1,3] × 10^−3^ mm^2^/s of *D* values that are reported for human brain studies [4, 23]. In abdominal organs the range extends up to 5 × 10^−3^ mm^2^/s [7]. In the text to follow we note that (i) the units associated with *D* and *b* have been omitted for readability (all *b*-values are stated in s/mm^2^) and (ii) $E\left(x\right)=\left(1/N_{\text{MC}}\right)\sum_{i=1}^{N_{\text{MC}}}\hat{x}$. It is also noteworthy that we used LSE throughout the paper for parameter estimation.

### 3.1. Comparison to GCRLB

**Figure 2:**
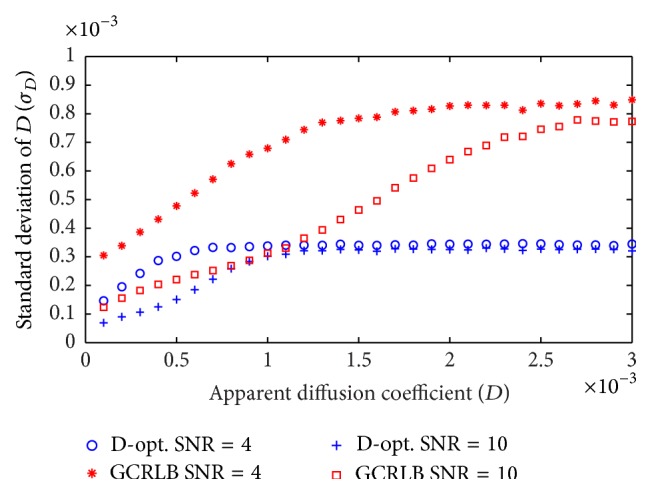
Standard deviation of estimated ADC values (*σ*_*D*_) for a range of *D* values, where *N* = 2, *b*_min_ = 0, *b*_max_ = 1500, *m*_0_ = 500, *N*_MC_ = 20000, and SNR = *m*_0_/*σ*_*G*_. The proposed D-optimal method is compared to GCRLB [23].

Given that the GCRLB method [23] is specifically designed for ADC estimation and is in good agreement with previous studies [21, 22], herein we compare the proposed D-optimal design with the GCRLB method.  Figure 2 shows the standard deviation of estimated ADC values (*σ*_*D*_) for a range of *D* values, where *N* = 2, *b*_min_ = 0, *b*_max_ = 2000, *m*_0_ = 500, *N*_MC_ = 20000, and SNR = *m*_0_/*σ*_*G*_. According to table 3 in [23] the optimal two-point design for *D* ∈ [0.1,3] × 10^−3^ is *b*_1_ = 0, *b*_2_ = 820 while the D-optimal method suggests *b*_1_ = 0, *b*_2_ = *b*_max_. Several key observations can be drawn from Figure 2. (i) Increasing the SNR from 4 to 10 significantly improves the performance of the GCRLB. In addition, the performance of the GCRLB is heavily dependent on the *D* values to be measured. In contrast, the performance of the D-optimal design is very consistent, demonstrating robustness to changes in SNR and *D*. (ii) The D-optimal design outperforms the GCRLB over the entire range of *D* values and SNRs. (iii) For small *D* values and high SNR, where the Rician distribution can be fairly approximated by a Gaussian distribution [25], the performance of the GCRLB is close to that of the D-optimal design. (iv) Though the Rician noise model does not match our theoretical noise assumption, the precision of $\hat{D}$ is independent of its actual value, *D*.

**Figure 3:**
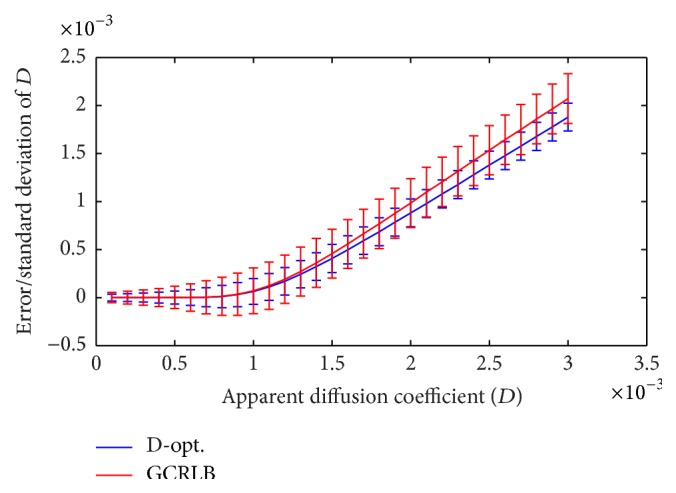
Error and standard deviation of estimated ADC values (vertical bars) for a range of *D* values, where *N* = 10, *b*_min_ = 0, *b*_max_ = 1500, *m*_0_ = 500, *N*_MC_ = 20000, and SNR = *m*_0_/*σ*_*G*_ = 10. The proposed D-optimal method is compared to GCRLB [23].

**Figure 4:**
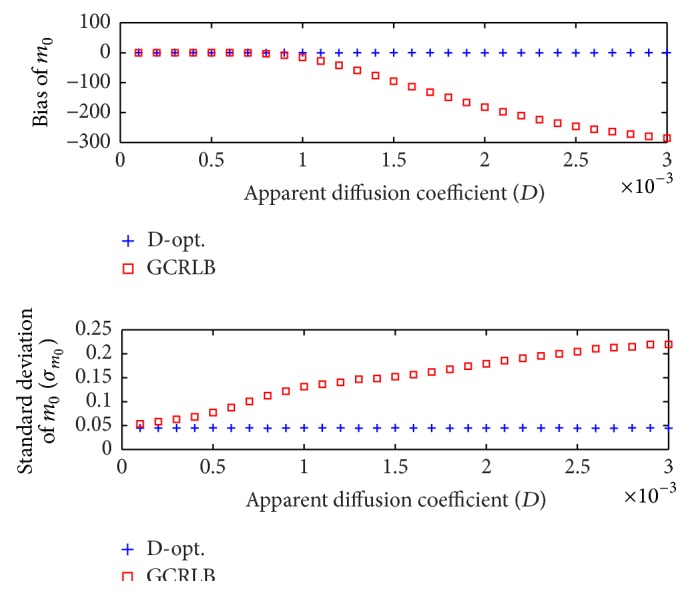
Bias and standard deviation of the estimated *m*_0_ values for a range of *D* values, where *N* = 10, *b*_min_ = 0, *b*_max_ = 1500, *m*_0_ = 500, *N*_MC_ = 20000, and SNR = *m*_0_/*σ*_*G*_ = 10. The proposed D-optimal method is compared to GCRLB [23].

Figure 3 compares the GCRLB and D-optimal designs in terms of error (computed as $\left|E\left(\hat{D}\right)-D\right|$) and standard deviation of $\hat{D}$ (illustrated as vertical bars for each *D* value), where *N* = 10, *b*_min_ = 0, *b*_max_ = 2000, *m*_0_ = 500, *N*_MC_ = 20000, and SNR = *m*_0_/*σ*_*G*_ = 10. According to table 3 in [23] the optimal ten-point design for *D* ∈ [0.1,3] × 10^−3^ is *b*_1_ = *b*_2_ = 0, *b*_3_ = *b*_4_ = ⋯ = *b*_8_ = 700, and *b*_9_ = *b*_10_ = *b*_max_ while the D-optimal design suggests *b*_1_ = ⋯ = *b*_5_ = 0, *b*_6_ = ⋯ = *b*_10_ = *b*_max_. It can be seen in Figure 3 that the D-optimal design performs better in terms of accuracy and precision.  Figure 4 shows, for the same test, plots of the bias (computed as $E\left({\hat{m}}_{0}\right)-m_{0}$) and standard deviation of ${\hat{m}}_{0}$. It can be seen that the proposed method leads to both accurate and precise estimation of *m*_0_. In addition, the D-optimal design shows very consistent performance over the whole range of *D* values. It is noteworthy that the GCRLB method severely underestimates *m*_0_ for high *D* values.

### 3.2. Sensitivity Analysis and Comparison to the ED Design

In this section we evaluate the sensitivity of the D-optimal design to changes in input parameters such as *D*, *N*, *b*_max_, and SNR. The input variable *b*_min_ is not considered because it is usually set to *b*_min_ = 0.

**Figure 5:**
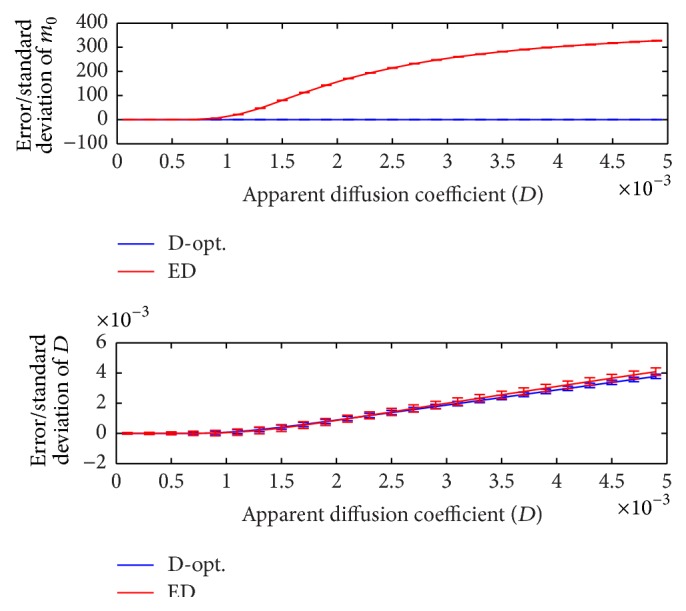
Error and standard deviation of $\hat{D}$ and ${\hat{m}}_{0}$ for the range *D* ∈ [0.1,5] × 10^−3^, where *N* = 10, *b*_min_ = 0, *b*_max_ = 2000, *m*_0_ = 500, *N*_MC_ = 20000, and SNR = *m*_0_/*σ*_*G*_ = 10. The D-optimal design is compared to the ED design (where *b*_*i*_s are equidistantly distributed between *b*_min_ and *b*_max_).

The ADC value may vary depending on tissue type, pathological/developmental changes, and aging. The error and standard deviation of $\hat{D}$ and ${\hat{m}}_{0}$ are illustrated in Figure 5 for the range *D* ∈ [0.1,5] × 10^−3^, where *N* = 10, *b*_min_ = 0, *b*_max_ = 2000, *m*_0_ = 500, *N*_MC_ = 20000, and SNR = *m*_0_/*σ*_*G*_ = 10. It can be seen that the D-optimal design outperforms the ED design in terms of accuracy and precision. Notably, the difference in estimation of *m*_0_ is extremely large. This can have a significant impact on studies that use the diffusion signal itself as a biomarker (as in [14]). Note that, for the D-optimal design, the variance of *σ*_*D*_ is almost fixed for *D* ∈ [1,5] × 10^−3^. This consistency of performance is also apparent in the estimation of *m*_0_.

**Figure 6:**
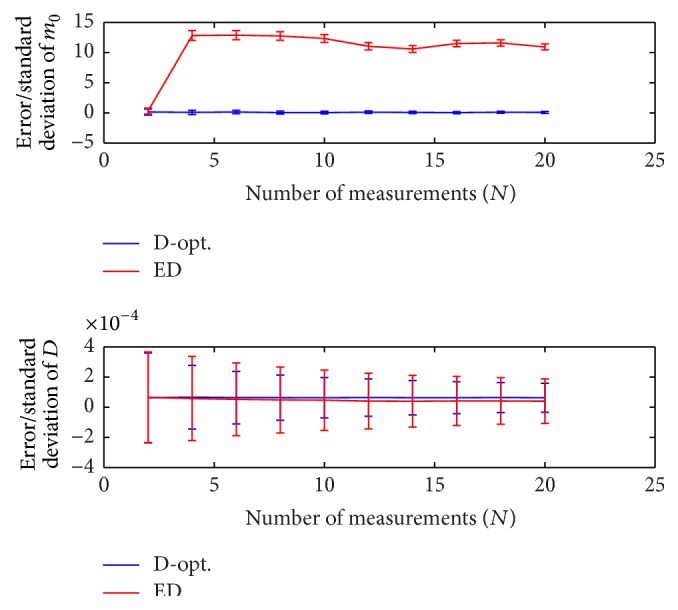
Error and standard deviation of $\hat{D}$ and ${\hat{m}}_{0}$ for the range *N* = 2 to *N* = 20, where D = 1 × 10^−3^, *b*_min_ = 0, *b*_max_ = 2000, *m*_0_ = 500, *N*_MC_ = 20000, and SNR = *m*_0_/*σ*_*G*_ = 10. The D-optimal design is compared to the ED design.

The number of measurements is in general limited by the available clinical scan time. Here, we consider a range of *N* that is feasible for clinical studies according to the literature. The error and standard deviation of $\hat{D}$ and ${\hat{m}}_{0}$ are illustrated in Figure 6 for the range *N* = 2 to *N* = 20, where *D* = 1 × 10^−3^, *b*_min_ = 0, *b*_max_ = 2000, *m*_0_ = 500, *N*_MC_ = 20000, and SNR = *m*_0_/*σ*_*G*_ = 10. It shows that the D-optimal design outperforms the ED design in terms of standard deviation (vertical bars) of $\hat{D}$ and ${\hat{m}}_{0}$. As *N* increases, the variance of the estimated parameters decreases for both design methods while the accuracy is almost constant. Note that for *N* = 2 the two design methods lead to the same solution. Deviating from D-optimal solution, accuracy/precision of ${\hat{m}}_{0}$ for the ED method considerably decreases even with higher number of measurements (cf. *N* = 2 with *N* = 4).

**Figure 7:**
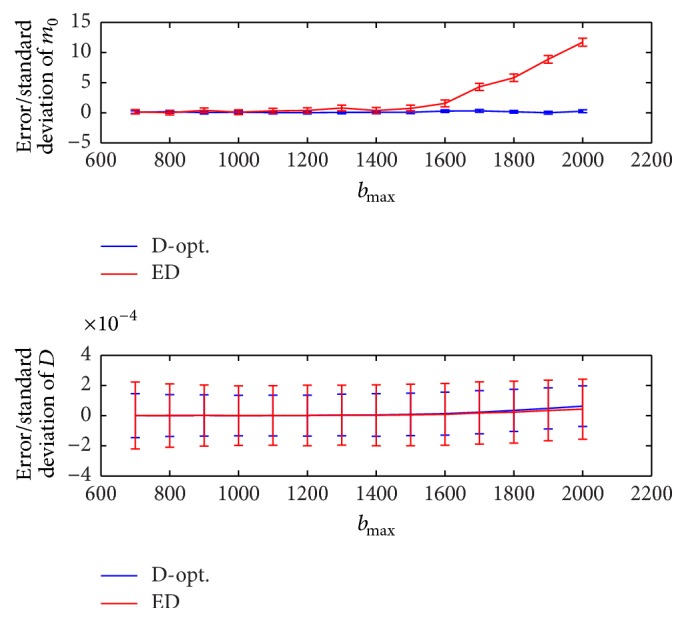
Error and standard deviation of $\hat{D}$ and ${\hat{m}}_{0}$ as a function of *b*_max_, where *D* = 1 × 10^−3^, *N* = 10, *b*_min_ = 0, *m*_0_ = 500, *N*_MC_ = 20000, and SNR = *m*_0_/*σ*_*G*_ = 10. The D-optimal design is compared to the ED design.

Different *b*_max_ values are recommended in the literature for different target organs/tissues. For example, [1] suggests *b*_max_ = 700 for kidney while *b*_max_ = 1500 is used for head and neck imaging [20] and *b*_max_ = 2000 for brain imaging [18]. The error and standard deviation of $\hat{D}$ and ${\hat{m}}_{0}$ are illustrated in Figure 7 for the range *b*_max_ = 700,800,…, 2000, where *D* = 1 × 10^−3^, *N* = 10, *b*_min_ = 0, *m*_0_ = 500, *N*_MC_ = 20000, and SNR = *m*_0_/*σ*_*G*_ = 10. It can be seen that the D-optimal design outperforms the ED design in terms of standard deviation (vertical bars) of $\hat{D}$ and ${\hat{m}}_{0}$. For the ED design, as *b*_max_ increases it does not make a considerable difference to the estimation of *D* but it does negatively impact on the estimation of *m*_0_ producing increasingly larger errors for values of *b*_max_ beyond 1600. In contrast, the D-optimal design shows a better and relatively consistent performance over the whole range (with almost constant accuracy and precision).

**Figure 8:**
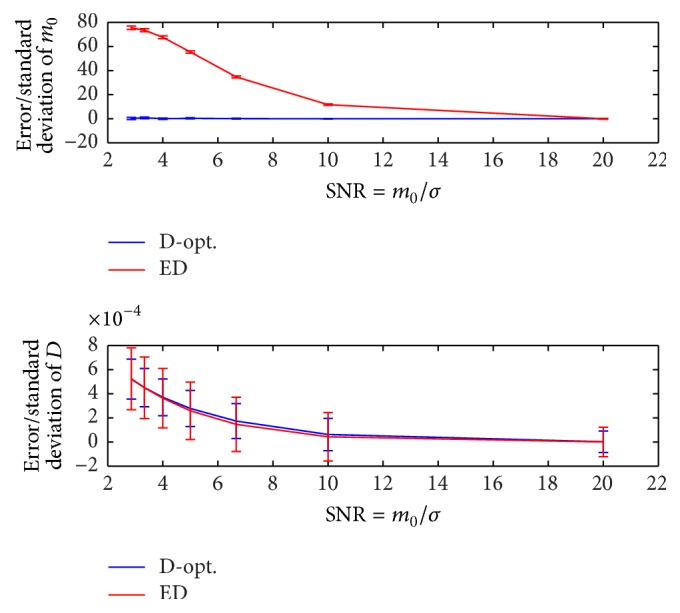
Error and standard deviation of $\hat{D}$ and ${\hat{m}}_{0}$ as a function of SNR (defined as *m*_0_/*σ*_*G*_), where *D* = 1 × 10^−3^, *N* = 10, *b*_min_ = 0, *b*_max_ = 2000, *m*_0_ = 500, and *N*_MC_ = 20000. The D-optimal design is compared to the ED design.

The signal-to-noise ratio also affects the accuracy and precision of the estimation problem. We investigate the effect of SNR on the proposed experiment design as follows. The error and standard deviation of $\hat{D}$ and ${\hat{m}}_{0}$ are illustrated in Figure 8 as a function of SNR (defined as *m*_0_/*σ*_*G*_), where *D* = 1 × 10^−3^, *N* = 10, *b*_min_ = 0, *b*_max_ = 2000, *m*_0_ = 500, and *N*_MC_ = 20000. It shows that, over the whole range of SNR values, the D-optimal design leads to minimum variance estimation of the unknown parameters. For ED design, the accuracy and precision of both $\hat{D}$ and ${\hat{m}}_{0}$ improve with increasing SNR while for the D-optimal design an improvement is only seen for $\hat{D}$.

### 3.3. Tests on a Mean Diffusivity Map

**Figure 9:**
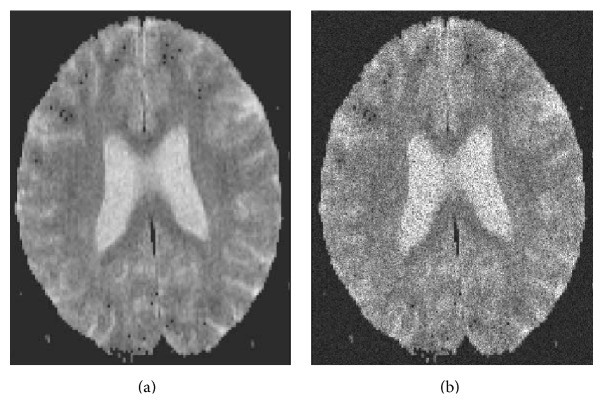
Original mean diffusivity map considered as ground truth (a) and an example noisy image produced by adding Rician distributed noise, where SNR = 5 (b). The original image is taken from [18].

To illustrate the impact that experiment design may have on diffusion-weighted images, we perform the following test. We take figure  4.(a) in [18] as the ground truth image. Then we add Rician distributed noise pixelwise (SNR = 5). The original image and an example of noisy images are shown in Figure 9.

**Figure 10:**
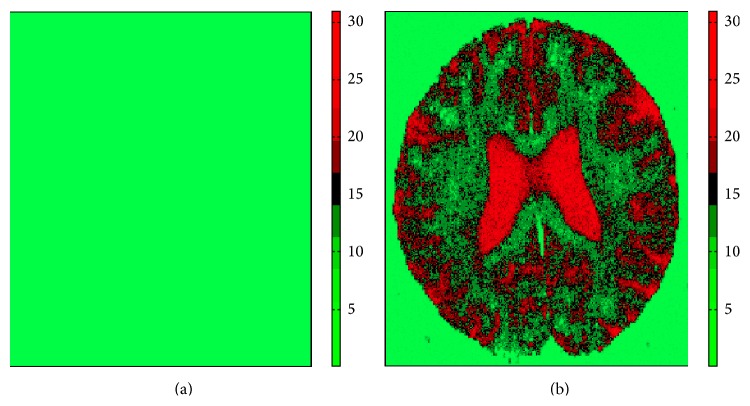
Error in estimation of diffusion signal computed as $\left|E\left({\hat{m}}_{0}\right)-m_{0}\right|$ for the D-optimal (a) and ED (b) design methods. The color bar ranges from 0 to 31.

**Figure 11:**
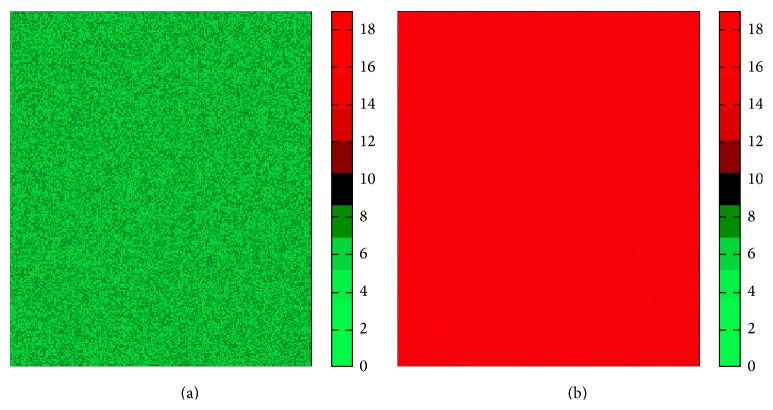
Standard deviation in estimation of ${\hat{m}}_{0}$. The D-optimal design (a) produces more precise estimates compared to the ED design (b). Note that the standard deviation is multiplied by 100 to enhance the visualization.

Let *I*(*i*, *j*) denote a pixel intensity in the original image. Then we run Algorithm 1 with the following setting for all pixels: *m*_0_ = *I*(*i*, *j*), SNR = *m*_0_/5, *N* = 20, *D* = 1 × 10^−3^, *b*_min_ = 0, *b*_max_ = 2000, and *N*_MC_ = 20000. The resultant images computed as error in estimation of ${\hat{m}}_{0}\;\;(\left|E\left({\hat{m}}_{0}\right)-m_{0}\right|)$ and its standard deviation are shown in Figures 10 and 11, respectively.  Figure 10 shows that one can accurately estimate diffusion signal using the D-optimal design. The accuracy is almost independent of the signal level. In contrast, the ED design produces large errors for high signal levels. In applications that consider statistics in small ROIs (such as [11]) this may be misleading.

The optimal experiment design is fundamentally based on improving the precision of the estimation problem. This can be seen in Figure 11, where we illustrate the standard deviation of ${\hat{m}}_{0}$. It can be seen that the D-optimal design consistently produces lower variance than the ED design. It is noteworthy that Figures 10 and 11 represent the sensitivity analysis with respect to *m*_0_ and confirm the stable performance of the D-optimal design.

## 4. Discussions

Although the current work focuses on ADC imaging, the proposed method can be applied in experiment design for other applications of the monoexponential model. As an example, empirical evaluations in [17] show that adding more measurements on *b*_min_ improves the results of model fitting in enzyme kinetics (compared to the ED design). This is in agreement with our findings.

Comparing the proposed method to previous studies, we see that it (i) is based on less restrictive noise assumptions and thus covers a wider range of applications, (ii) shows that the optimal design is not necessarily dependent on the imaged parameters, (iii) outperforms the ED and GCRLB methods (based on evaluations using simulated data), and (iv) is applicable even if the noise assumptions are partly violated.

Figures 3 and 5 show that (i) in contrast to the findings in [23] there exist *D*-independent optimal designs that minimize variance of the estimated ADC values and (ii) even using optimal designs *D* values greater than 1.1 × 10^−3^ cannot be estimated accurately. In other words, the error of $\hat{D}$ is larger than 10% when *D* > 1.1 × 10^−3^. This means that ADC values reported for cartilage, muscle [23], and normal white matter [4] are accurate/reliable while high ADC values reported for normal kidney [7] should arguably be treated with caution. This warrants further investigation using real data.

*Noise Distribution.* The noise assumptions (independency and equal variance) are necessary for tractable theoretical derivations but do not necessarily limit the proposed method to Gaussian noise. For example, these assumptions hold for scenarios with independent but nonidentical noise distributions provided that they have equal variances. We have evaluated the proposed method in realistic cases (independent Rician noise with nonequal variances). However, in modern scanners with phased arrays and multicoil acquisition [26], the noise is noncentrally *χ*-distributed [27]. The D-optimal design problem for such kind of noise distributions is intractable. In addition, relaxing the* “equal variance”* condition, the D-optimal design becomes dependent on the imaged parameters. Thus, we can not find an optimal design that performs well over the whole range of the imaged parameters.

*Estimation Method.* One can use other estimation methods instead of LSE. Possibilities include the median estimator [18], maximum likelihood estimator (MLE), and weighted least squares (WLS) [28]. The exact formulation of the D-optimal design problem for these estimation techniques is dependent on the noise distribution and often becomes intractable. In the case of Gaussian noise the MLE leads to the same solution as LSE.

*Physical Considerations.* Given that the proposed method takes the minimum and maximum *b*-values as the input, one can adjust the range to avoid the signal distortion caused by physical phenomena such as the perfusion effect at low *b*-values and non-Gaussian behavior at high *b*-values.

## 5. Conclusion

The need for precise estimation of biomedical quantities has given rise to studies concerning optimal experiment design for monoexponential model fitting. In this paper, we formulated the problem as a D-optimal design problem that is a convex optimization problem. In contrast to previous studies, we did not restrict our theoretical framework to model fitting in the presence of Gaussian noise. Solving this problem and evaluating the results for ADC imaging on simulated data, we showed that the optimal design is independent of the imaged parameters. Furthermore, Monte Carlo simulations showed that the D-optimal design outperforms the ED and GCRLB methods. Moreover the proposed method is applicable to a wider range of problems because of its less restrictive noise assumptions. Our evaluations show that it is applicable even if the noise assumptions are partly violated. An important practical result is that accurate estimation of high ADC values is not possible even using optimal experiment design.

## Figures and Tables

**Algorithm 1:**
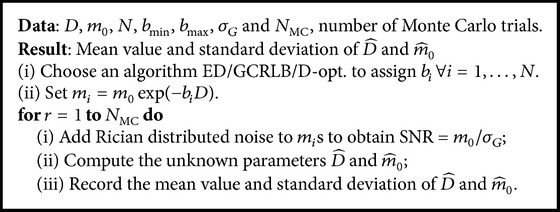
Pseudo-algorithm to evaluate an experiment design.

## Appendices

### A. Evaluation of an Experiment Design

Here we provide a pseudo-code for the algorithm used in Section 3 (see Algorithm 1).

### B. GCRLB Experiment Design

In an estimation problem, the lower bound of the variance of a parameter *x*_*j*_, *σ* ^2^(*x*_*j*_), is given by the corresponding diagonal element of the inverse of the Fisher information matrix:

(B.1) $$\begin{array}{c} \sigma^{2}\left(\;x_{j}\;\right)\geq {\left(\;F^{-1}\;\right)}_{jj}. \end{array}$$

This is known as the Cramer-Rao lower bound (CRLB). Assuming zero-mean Gaussian noise on *m* in (1), one can obtain the following Fisher information matrix for ADC imaging [23]:

(B.2) F=1σG·∑i=1Nexp⁡−2biD−∑i=1NbiS0exp⁡−2biD−∑i=1NbiS0exp⁡−2biD∑i=1Nbi2S02exp⁡−2biD.

In the derivation above $x=\left[\begin{array}{cc} S_{0} & D \end{array}\right]$. The GCRLB experiment design entails minimizing the CRLB of *D*, (**F** ^−1^)_22_, with respect to *b*_*i*_s for a given value/range of *D*. The reader is referred to [23] for more details.
